# The Week's Work

**Published:** 1909-10-09

**Authors:** 


### The Week's Work.

The report of the Board of Superintendence of the eleven Dublin city hospitals which received Parliamentary grants for the year ending March 31 is now issued. While commenting highly on the management, economy, and treatment in these institutions, the Board finds fault with the out-patient departments. The Board, however, is of opinion that want of funds alone accounts for this: "The state of the intern departments is proof of the capabilities and good-will of the hospital authorities, and had they funds no doubt all parts of the institution under their control would be on an equal level." Since two classes of patients come to the out-patient department, those seeking admission to the wards, besides the out-patients proper, the want of funds which the Board says characterises each institution affects a very large number of sufferers. The total income for the eleven hospitals was £63,493, and the maintenance and establishment expenditure was £56,766. In all 14,710 patients were treated throughout the year.

The recent discussions on the Finance Bill in the House of Commons during the week have had a special interest for those responsible for the finances of the voluntary charities, especially hospitals. We are glad to see that Mr. Balfour called Mr. John Massie sharply to account for stating that the motive of the large benefactor to universities was to erect some monument, not to learning, but to his own benefit. Mr. Balfour was right in censuring the terms of contempt expressed for those who leave their money to such institutions. Mr. Massie, it is true, in the *Times* of the 30th ultimo, disclaims any conscious intention of the motives attributed to him, but he makes the matter worse by extending his remarks to all benefactions. He declares that bequests for what are termed charitable purposes are "often useless and deleterious bequests, gifts, as is well known, frequently deferred till the money is of no further use to the owner."

Formerly, it is possible that many institutions, including the voluntary hospitals, did benefit by benefactions from men who took a great interest in their management, and largely controlled it during their lifetime. Nowadays the voluntary hospitals are placed upon a much wider basis. Seeing that there is no purpose so free from objection as the work of a voluntary hospital, Mr. Massie's criticism cannot apply to a hospital. We have a right to expect that members of Parliament will display judgment and care in dealing with vital principles, upon the enforcement of which the welfare of large masses of the people may depend. One such principle is the voluntary support of hospitals, which has produced, on the whole, the most perfect system of administration and the most progressive development in treatment and management at our hospitals which is to be found anywhere in the civilised world. We would advise Mr. Massie to study the question in all its bearings, and so to attempt to understand it, before he ventures again to expose himself to the just and forcible criticism of leaders of public thought, and of all workers for the sick throughout the Empire.

During the past week a good deal of attention appears to have been excited in various directions amongst the hospitals in regard to laundries and their work. Mr. Stallard, the Secretary of the Worcester General Infirmary, writes to inquire whether there is a hospital with an up-to-date laundry within easy reach of Worcester. We would recommend him to go to the Coventry and Warwickshire Hospital, where he will find an up-to-date laundry, admirably planned and efficiently worked, which is entitled to be regarded as a type of what a hospital laundry should be. Mr. Stallard also seeks information as to the best course to pursue in removing foul linen from the wards. We published an article on this subject in The Hospital of June 6, 1908, page 271. Here we may state that the galvanised iron boxes used at the Leicester Infirmary are a good pattern for hospital use. Some hospitals have linen shoots to secure the ready removal of foul linen, which consist of iron chambers or tubes which are open to the air from top to bottom. It is probable that the galvanised iron trolley will be found more convenient, and on the whole the best appliance for this purpose.

Most people nowadays connected with hospitals take the warmest interest in the progress of the League of Mercy. As the result of ten years' organisation it has secured the voluntary services of something like 16,000 workers, who have succeeded in interesting between three and four hundred thousand associates. The great difficulty in an organisation of this character is to prevent wastage through the retirement of vice-presidents and members. What this wastage amounts to will be realised when it is stated that in the twelve months ending September 1909, 93 vice-presidents resigned and 154 new vice-presidents were appointed, showing a net increase for the year of 61. We could wish that it were possible to gather together every year those who retire from the League of Mercy in order that they may have an opportunity of stating their reasons for withdrawal. If such a Conference were instituted, we believe that the majority of those retiring would reconsider their decision, and continue to render, with alacrity, a measure of personal service in the days of health in the cause of the sick.

According to the local paper, the Blaby Guardians have decided to instruct their medical officer that people "who meet with an accident, and who were likely to become chargeable to the parish, should be sent to the infirmary whenever possible." This resolution has been evoked by several bills for medical attention on people not in receipt of out relief. Commenting upon this resolution, our contemporary remarks: "One can scarcely be surprised that the Board should determine to keep down the rates by this and other methods. On the other hand, the subscribers to Leicester Infirmary may fairly protest against their charity being thus unexpectedly diverted to this purpose, more especially those in the borough, who are already rated, and heavily, to maintain an infirmary of their own. If the Blaby Guardians really think that all their serious local accidents should be treated in Leicester Infirmary, the least they can do is to forward an annual subscription sufficient to cover all extra expense thus entailed, instead of shouldering the entire cost on the philanthropic." With which we thoroughly agree.

Mr. Max Pemberton writes to the *Times* drawing attention to the fact that the King proposes to visit the City of Norwich upon October 25, and it is hoped that his Majesty will then lay the foundation-stone of the new buildings connected with the Norfolk and Norwich Hospital. Dealing with the hospital, he remarks that it is an institution which should make a wide appeal to others than the people of a city which educated Nelson and has harboured so many men of literary and scientific distinction. There are but 410 beds in the hospitals at Norfolk, and of these 210 are in the building which the "Man of Shottesham" founded 140 years ago. "Norfolk," he writes in conclusion, "is a county which, unhappily, abounds in cases of cancer and of other malignant diseases attributed in a large measure to the marshy character of the lowlands. But it is also a county beloved by the holiday-maker, and fruitful in gifts of health beyond almost any other to the many thousands who frequent its coasts and waters in the summer. To these it is hoped that this appeal may go out, and that all who love East Anglia will of their gratitude make some just return to a people and a county which minister so surely to the pleasures of so many." It would be a good thing for our hospitals if other popular writers will manifest as intelligent and as active an interest in our charitable institutions as the author of "The Iron Pirate" does.

Mayor McClellan's Commission, appointed as far back as 1906 to study the organisation of the New York Public Hospitals, has just issued its final report. The Commission appears to have gone very fully into the question of hospital administration as at present existing in the commercial capital of the United States, and to have arrived at some very interesting conclusions. Thus it practically unanimously recommends the creation of a special "Department of Public Hospitals." This department is to be administered by a Special Commissioner of Public Hospitals, who will be the virtual supreme head of the hospitals in New York City, and who will have under his charge the entire public hospital and ambulance service. The Commissioner is to be a medical man of at least 10 years' standing, who has had institutional experience and is possessed of business ability. He is to receive a salary of not less than £2,000 a year, and is to hold office for a term of 10 years. The department he controls is to have absolute charge of all existing city hospitals, sanatoria, infirmaries, and ambulance relief stations, with authority to extend the hospital system as rapidly as means are granted for that purpose by the establishment of new institutions. The Commission, it is satisfactory to see, considers that while hospitals are there primarily and principally for the sake of the sick, it is urgently necessary that the existing rights of medical educational institutions and medical boards should be carefully safeguarded.

Dealing with the matter of ambulance relief work, the Commission makes some practical suggestions which are worth consideration. It advises that the whole service should be in charge of the Special Commissioner, and should be considered an integral part of his department. This department must possess power to make appropriate contracts with private hospitals for full co-operation in the service, to provide the necessary clinical force to maintain the service, and to create an ambulance service which should be efficient, trustworthy, and a credit to the city. A very excellent suggestion which we think might be adopted by the London City authorities is to the effect that the departmental ambulances are to have the same right of way as is now accorded to fire engines, but that no ambulances other than departmental vehicles are to have such right of way. All calls are to be repeated by the department, immediately upon receipt, to police headquarters to enable the sending of a police officer to the point of call. A special service is to be organised for the transfer of patients from one hospital to another and for the carrying of non-urgent cases from private houses to hospitals. Such special ambulances are to call upon any patient upon the request of a hospital, and to transfer such patient to the hospital making the call or to any other institution.

There has been, as was to have been expected, considerable discussion on these points. If the Commission's suggestions are carried out wholeheartedly, most of the professional papers on the other side agree that New York City will possess a comprehensive and uniform hospital and ambulance system which will be second to none in the world. The public hospitals of Paris, with a capacity of nearly 15,000 beds, are under a somewhat similar central department, while other Continental cities which have adopted a central system, though not in its entirety, are Berlin, Copenhagen, Vienna, Hamburg, Munich, and Brussels. In the United States the city of St. Louis has already initiated some such scheme as the Commission advocates, and it is working successfully. New York, with its enormous and rapidly increasing population, is behind Berlin and Paris in the matter of bed provision in its public hospitals, but it is satisfactory to note that there is to be a large increase in the number of beds in most of the new institutions, and that newer ones are being built or planned.

Considerable discussion has been evoked by the Commission's recommendation that the head of the new department should be a medical man of 10 years' standing. From this recommendation one of the medical members of the Commission, Dr. Darlington, dissents. From a purely professional point of view it would be in the best interests of the medical men of a large city that the departmental head of the hospitals should be a medical man of experience and broad-minded, liberal views, but it may be doubted whether it would be in the best interests of the public as well. That medical superintendents of hospitals are necessary and excellent no one who has followed their work in the past can have any reasonable doubt, but it is permissible, even when one is fully prepared to admit that as hospital superintendents medical men have done excellently well, to question the advisability of putting a man who, with all his broadmindedness and business capacity, is the trained product of one hospital and the student of one school in supreme command over a heterogeneous collection of institutions each with individual and separate interests. Such a chief should, of course, have his medical advisory board; but in our opinion there is much to be said in favour of giving the appointment to a well-trained layman who has had experience of hospital work. That has been done in Paris with excellent results, and to some extent in Berlin.

